# Polypharmacy Is Significantly and Positively Associated with the Frailty Status Assessed Using the 5-Item FRAIL Scale, Cardiovascular Health Phenotypic Classification of Frailty Index, and Study of Osteoporotic Fractures Scale

**DOI:** 10.3390/jcm10194413

**Published:** 2021-09-26

**Authors:** Chi-Di Hung, Chen-Cheng Yang, Chun-Ying Lee, Stephen Chu-Sung Hu, Szu-Chia Chen, Chih-Hsing Hung, Hung-Yi Chuang, Ching-Yu Chen, Chao-Hung Kuo

**Affiliations:** 1 Department of Occupational and Environmental Medicine, Kaohsiung Municipal Siaogang Hospital, Kaohsiung Medical University, Kaohsiung City 812, Taiwan; ciem273555@gmail.com; 2 Department of Occupational and Environmental Medicine, Kaohsiung Medical University Hospital, Kaohsiung Medical University, Kaohsiung City 807, Taiwan; ericch@kmu.edu.tw; 3 Department of Family Medicine, Kaohsiung Municipal Siaogang Hospital, Kaohsiung Medical University, Kaohsiung City 812, Taiwan; 4 Department of Family Medicine, Kaohsiung Medical University Hospital, Kaohsiung Medical University, Kaohsiung City 807, Taiwan; june.lee@gap.kmu.edu.tw; 5 Department of Dermatology, Kaohsiung Municipal Siaogang Hospital, Kaohsiung Medical University, Kaohsiung City 812, Taiwan; stephenhu30@hotmail.com; 6 Department of Internal Medicine, Kaohsiung Municipal Siaogang Hospital, Kaohsiung Medical University, Kaohsiung City 812, Taiwan; scarchenone@yahoo.com.tw (S.-C.C.); kjh88kmu@gmail.com (C.-H.K.); 7 Environmental and Occupational Medicine Center, Kaohsiung Municipal Siaogang Hospital, Kaohsiung Medical University, Kaohsiung City 812, Taiwan; pedhung@gmail.com; 8 Department of Family Medicine, National Taiwan University Hospital, Taipei City 100, Taiwan; cycchen@ntu.edu.tw

**Keywords:** polypharmacy, frailty, Cardiovascular Health Study, Study of Osteoporotic Fracture

## Abstract

The aim of this study was to investigate the association between frailty and polypharmacy using three different frailty screening tools. This was a cross-sectional study of people aged ≥65 years. Participants were included and interviewed using questionnaires. Polypharmacy was defined as the daily use of eight or more pills. Frailty was assessed using a screening tool, including (1) the Fatigue, Resistance, Ambulation, Illness and Loss of Weight Index (5-item FRAIL scale), (2) the Cardiovascular Health Phenotypic Classification of Frailty (CHS_PCF) index (Fried’s Frailty Phenotype), and (3) the Study of Osteoporotic Fracture (SOF) scale. A total of 205 participants (mean age: 71.1 years; 53.7% female) fulfilled our inclusion criteria. The proportion of patients with polypharmacy was 14.1%. After adjustments were made for comorbidity or potential confounders, polypharmacy was associated with frailty on the 5-item FRAIL scale (adjusted odds ratio [aOR]: 9.12; 95% confidence interval [CI]: 3.6–23.16), CHS_PCF index (aOR: 8.98; 95% CI: 2.51–32.11), and SOF scale (aOR: 6.10; 95% CI: 1.47–25.3). Polypharmacy was associated with frailty using three frailty screening tools. Future research is required to further enhance our understanding of the risk of frailty among older adults.

## 1. Introduction

Frailty is a clinical syndrome characterized by physical activities, cognition and emotional impairment [1]. It is a state of increased vulnerability to stressors, especially in older adults [1], that leads to adverse health outcomes such as falls, disability, hospitalization, institutionalization and mortality [2,3,4]. As people of advanced age are gradually increasing in many countries, frailty is considered of growing importance in geriatric population. Previous studies estimated that the prevalence of frailty varied from 3.9% to 51.4% [5,6,7]. This variance is influenced by the differences in countries and socioeconomic status and, more importantly, screening tools. Because there is no gold standard for assessing frailty, multiple assessment methods have been developed, such as Fried’s phenotype model [8], and the frailty index of Rockwood’s cumulated deficit model [9]. These screening tools help to identify frail patients at high risk for negative health outcomes and provide an opportunity to prevent the progression of comorbidity [10].

Polypharmacy refers to multiple use of medications and/or unnecessary drug use, commonly defined by five or more medications daily [11,12]. A systematic review [11] reported that even if the most common definition of polypharmacy was the numerical definition of five or more medications daily (*n* = 51, 46.4% of articles), some articles [13,14] use definitions with ten or more medicines. Polypharmacy is common in older adults especially with multimorbidity, as one or more medications may be used in each condition. The patient may suffer from increased risk of various adverse effects and harm. A retrospective study demonstrated that larger numbers of medications used among 259 patients attending a cardiovascular outpatient department were associated with faster declines in renal function and may have worsened the prognosis [15]. Additionally, potential adverse outcomes included declines in cognition and mobility as well as drug-drug interactions and drug-disease interactions [16,17].

Since both frailty and polypharmacy have been considered as common geriatric syndromes, they have gained increasing interest and attention. However, the association between frailty evaluation and polypharmacy is still scarce. Therefore, we aim to investigate the association between frailty and polypharmacy.

## 2. Materials and Methods

### 2.1. Subjects

The study was a cross-sectional study. Participants were included, after informed consent, from the outpatient clinic and the health examination department in a single center located in the urban area of Kaohsiung City. The inclusive criterion was adults aged 65 years old or more. The exclusive criteria were (1) less than 65 years old, (2) mental disability or psychological disease, and (3) who were unwilling to agree to the informed consent form or were unable to cooperate with the study. During October 2020 to April 2021, 205 participants were included. The IRB number was KMUHIRB-F(I)-20200052.

### 2.2. Measurement and Questionnaire

Participants were interviewed by questionnaire. One-on-one interviews were conducted by well-trained interviewers. Participants could complete the questionnaire with the assistance of interviewers or their family. The demographic characteristics included gender, age, living environment, education level, and smoking and alcohol habits. Low education was defined as elementary school or no education. Daily use of more than eight pills was consider as polypharmacy. Evaluation tools for frailty included (1) Fatigue, Resistance, Ambulation, Illness and Loss of Weight Index (FRAIL model) [18]; (2) Cardiovascular Health Phenotypic Classification of Frailty index (CHS-PCF, Fried’s Frailty Phenotype) [19]; and (3) Study of Osteoporotic Fracture Index (SOF) [20]. All questionnaires were input into the computer twice by two independent personnel and the input correction was checked.

### 2.3. Frailty Evaluation

The 5-item FRAIL scale represented the abbreviation of Fatigue, Resistance, Ambulation, Illness, and Loss of weight, which was established at the International Nutrition, Health and Ageing Group. The scale consists of five items: (1) exhaustion, (2) weakness, (3) slowness while walking, (4) low activity, and (5) weight loss. These factors represent biological factors (fatigue and weight loss), functional factors (resistance and ambulation), and deficit accumulation by illness. The 5-item FRAIL scale classification was defined as frail (3–5), pre-frail (1–2), and robust health status (0) [18].

Fried’s phenotype model of frailty is also known as the Cardiovascular Health Phenotypic Classification of Frailty (CHS_PCF) index, a biological model of frailty comprising five components: unintentional weight loss, feelings of exhaustion, decreased physical activity, slow walking speed, and weakness. CHS_PCF scores were categorized as frail (3–5), pre-frail (1–2), and robust health status (0) [8,19].

The Study of Osteoporotic Fractures (SOF) scale comprised two factors with three components, including biological factors (inability to complete five chair rises, weight loss) and functional factors (reduced energy level). SOF scale scores were categorized as frail (2–3), pre-frail (1), and robust health status (0) [20].

### 2.4. Statistics

Descriptive statistics were used to analyze the means and dispersion of the continuous variables, including the age and the scores of the three frailty assessment tools. For the categorical variables, such as gender and smoking and alcohol consumption, numbers and proportions were used for this evaluation. Furthermore, we divided the participants into different groups according to different frail conditions. The 5-item FRAIL scale was divided into robust health, pre-frail and frail groups. CHS_PCF was classified into robust health, prefrail, and frail groups. SOF was divided into robust health, pre-frail and frail groups. Analysis of variance was performed to analyze the differences in polypharmacy among the frail groups. The non-frail groups (robust health and pre-frail participants) were selected as a reference. The crude logistic regression model of frail condition and polypharmacy, or frail condition and other variables including age, body mass index (BMI), gender, education level, living situation, and smoking and alcohol habits were performed. Finally, two adjusted logistic regression models (one by the Hosmer–Lemeshow goodness-of-fit test and the other by the exact logistic regression model) were conducted. IBM-SPSS version 20 statistical software (IBM Corp. Released 2011. IBM SPSS Statistics for Windows, Version 20.0. Armonk, NY, USA: IBM Corp.) was used, with a significant *p* value of 0.05, two-tailed tests.

## 3. Results

During the period of October 2020 to April 2021 we had registered 400 participants, of whom 205 fulfilled our inclusion criteria. Table 1 shows key descriptive analysis of the study baseline. We classified these participants into robust health, pre-frail, and frail groups by the 5-item FRAIL scale, CHS_PCF, and SOF scale according to these scale definitions, respectively. In FRAIL model, 105 (51.22%) were robust health, 54 (26.34%) were pre-frail, and 46 (22.43%) were frail; in CHS_PCF, 38 (18.53%) were robust health, 73 (35.61%) were pre-frail, and 94 (45.85%) were frail; in SOF model, 138 (67.31%) were robust health, 54 (26.34%) were pre-frail, and 13 (6.34%) were frail. Detailed demographic data on the different scales is shown in Table 1. Of these 205 participants, their average age was 71.1 years and average BMI was 26.06 kg/m^2^. The participants included 95 males (46.3%) and 110 females (53.7%). Moreover, 42.9% (88 participants) of all participants were of low education (elementary school only or no education), 18% (37 participants) lived alone, 4.4% (nine participants) were smokers, 8.8% (18 participants) consumed alcohol and 14.1% (29 participants) were polypharmaceutical.

We defined these non-frail participants as the reference group and a logistic regression model was created to analyze the variance of differences and odd ratio in polypharmacy as well as other potential confounders between the frail group and the reference groups. Moreover, the regression model was adjusted by potential confounders, including age, BMI, gender, education level, whether or not patients lived alone, and smoking and alcohol habits, in case these potential confounders and frailty revealed a significant association in the crude regression model. Comorbidity was considered as an important risk factor of frailty, so we adjusted it in the adjusted regression model. Table 2 shows that in the crude regression model of the FRAIL scale, the factor most strongly associated with 5-item FRAIL scale frail status was polypharmacy (odd ratio [OR] 10.49, 95% confidence interval [CI] 4.40–24.99, *p*-value < 0.001). Otherwise, parameters such as age, BMI, gender, education level, living environment, and smoking and alcohol habits were not associated with frailty in 5-item FRAIL scale. Furthermore, the adjusted model and the exact logistic regression shown a consistent trend; frailty was significantly and positively associated with polypharmacy after adjusting comorbidity (aOR * 9.12, 95% CI 3.6–23.16, *p*-value < 0.001; the exact logistic regression model aOR † 9.02, 95% CI 3.32–26.16, *p*-value < 0.001, respectively).

In Table 3, the crude regression model of CHS_PCF identified the factors, including polypharmacy (OR 13.76, 95% CI 4.01–47.23, *p*-value < 0.001), age (OR 1.07, 95% CI 1.02–1.13, *p*-value 0.010) and low education level (high education versus low education OR 0.46, 95% CI 0.26–0.80, *p*-value 0.007) which were associated with frail status in CHS_PCF model. Compared with participants with non-comorbidity, participants with three or more comorbidities were significantly and positively associated with frailty (OR 4.70, 95% CI 1.62–13.62, *p*-value 0.004). However, after adjusting for comorbidity, polypharmacy revealed consistent significantly positive association with CHS PCF frailty (aOR * 8.98, 95% CI 2.51–32.11, *p*-value < 0.001; aOR † 8.81, 95% CI 2.42–48.81, *p*-value < 0.001, respectively). Finally, the regression model of SOF in Table 4 determined that the factor associated with frailty was polypharmacy (crude OR 4.38, 95% CI 1.32–14.47, *p*-value 0.016; aOR * 6.10, 95% CI 1.47–25.3, *p*-value 0.013; and aOR † 6.31, 95% CI 1.21–33.29, *p*-value 0.027). Age, BMI, gender, education level, and living environment were not associated with frail status in the SOF model.

## 4. Discussion

To our knowledge, this is the first study comparing three different frailty models to examine the association between frailty and polypharmacy. This cross-sectional study explored the association of polypharmacy and frailty using the 5-item FRAIL scale, CHS_PCF index, and the SOF scale. The results showed that polypharmacy was strongly associated with frailty in each tool. Previous studies demonstrated similar results [21,22,23,24]. In Korea, Jung et al. [21] reported that frailty using the 5-item FRAIL scale was associated with the use of an increased number of medications. Furthermore, Jung et al. [22] reported that polypharmacy with five drugs or more was associated with physical frailty (OR 1.61, 95% CI 1.13–2.30) among 2907 adults aged 70–84 years in Korea, using the CHS_PCF index. In France, Herr et al. [23] reported that polypharmacy with five to nine drugs (OR 1.77, 95% CI 1.20–2.61) and excessive polypharmacy with ten drugs or more (OR 4.47, 95% CI 2.37–8.42) were associated with physical frailty among 2350 adults aged 70 years and over in France, also using the CHS_PCF index. Another cross-sectional study in France reported that polypharmacy with five drugs or more and inappropriateness of medications were both associated with the CHS_PCF index score in people aged 65 and over [24]. Additionally, an eight-year longitudinal cohort study which involved 4402 participants in North America using the SOF scale also demonstrated that using four to six medications had a higher risk of frailty of 55% (hazard ratio [HR] 1.55; 95%CI 1.22–1.96), and using more than seven drugs increased this to approximately 147% (HR 2.47; 95% CI 1.78–3.43) [25]. A systematic review evaluated the correlation between frailty and polypharmacy and demonstrated that frailty was associated with polypharmacy, especially with an increasing number of medications used; however, the definitions of frailty and polypharmacy varied between each article [26]. We used three tools for the frailty evaluation and found a consistent trend in our study which matched these previous publications.

Polypharmacy is strongly associated with multi-morbidity, since one or more medications may be used for each illness [27]. However, polypharmacy has potential adverse effects including falls, cognitive impairment, adverse drug events, increased health care utilization, hospitalization, and mortality [28,29]. The potential mechanisms of these adverse outcomes were multidimensional. As age increases, changes in metabolism may result in drug toxicity and drug-drug interaction [30]. Decreased body weight, decreased blood pressure and dehydration are common in older people [31,32,33]. Overtreatment by antihypertensive medicines and diuretics may cause hypotension or dehydration, which are associated with frailty, falls, syncope, poor cognition, disability and mortality [30].

Frailty results from an accumulation of multiple age-related health issues, and not surprisingly is associated with increased pharmaceutical intervention [34]. Since more and more importance is attached to inappropriate polypharmacy, determining interventions to improve appropriate polypharmacy is essential. Davies [29] reported that polypharmacy was significantly associated with both prefrailty (pooled OR 1.52; 95% CI 1.32–1.79) and frailty (pooled OR 2.62, 95% CI 1.81–3.79). However, it was also found that almost all reviews identified polypharmacy by medication count; few examined the relationship between disease states and drug groups. Another systematic review found that there were no studies examining the negative effects of single drugs on frailty, and some specific medications had benefits and improvements with respect to physical performance, muscle strength or body composition [35]. Nwadiugwu [34] stated that person-centered care for frail people may provide better engagement with individuals to reduce the occurrence of polypharmacy. Providing the frail patients with a comprehensive geriatric assessment and using medication prescription aid with Beers criteria and the STOPP/START criteria may decrease unnecessary medication usage and reduce the extent of frailty and polypharmacy [36,37]. Furthermore, alternative prescriptive interventions such as regular exercise and social participation could reduce medication use as well as treat frailty [38].

This study found that low education level was associated with frailty status in the CHS_PCF model. Previous studies have examined the impact of educational level on frailty [39,40]. In a Dutch 13-year longitudinal study, Hoogendijk et al. demonstrated that low education levels in older adults had a higher OR of being frail compared with those with a high educational level (relative index of inequality OR 2.94; 95% CI 1.84–4.71) [39]. Another study in Brazil investigated patients with limited formal education (defined by 0–4 years of formal schooling) and found that having no formal education increased the odds ratio of being categorized as frail (OR 2.0; 95% CI 1.0–3.9) [40]. Furthermore, limited education was also associated with cognitive performance and functional disability [40]. Similar results have been reported by several studies [41,42]. Explanations for educational effects on frailty may be based on shared associated factors such as socioeconomic differences and low income which can result in lack of access to healthy food, obesity and living in deprived neighborhoods [43,44].

Living alone was not associated with frailty in our study in any of the three models. Multiple previous studies have reported that living alone had adverse health effects, such as social isolation, loneliness, as well as depression [45,46]. However, living alone was barely considered a risk factor for frailty. A systematic review found that living alone is associated with physical frailty in cross-sectional studies but not significant in longitudinal studies; furthermore, men living alone are more likely to be frail than women [47]. In a cross-sectional study that investigated 1602 Japanese men and women living on isolated islands reported that living alone was associated with frailty in men (OR 3.85; 95% CI 1.94–7.65), but not in women (OR 1.08; 95% CI 0.72–1.63) [48]. Another cross-sectional study also demonstrated that living alone is associated with frailty in Hong Kong men, but not in Hong Kong women or Taiwanese people [49]. A potential explanation is that women are thought to have larger or more diverse social networks than men [50]. Social networks may be an important confounding factor that influences the effect of living alone and multiple adverse effects [51]. Our study was conducted in a Taiwanese urban area, and the relationship between living alone and frailty may be affected by social networks which depend on differences in local policy and culture.

There are several limitations in this study. First, this is a cross-sectional study that could only demonstrate associations and could not infer causality. Further longitudinal study is required for the demonstration of causality between polypharmacy and frailty. Second, we used self-reported questionnaires, and the results may be influenced by recall biases involving memory, mood or cognition. Finally, we specially investigated a single area in an urban locale, which might not present the whole picture of Taiwan.

## 5. Conclusions

This study demonstrated that polypharmacy is associated with frailty under the 5-item FRAIL scale, CHS_PCF index, and SOF scale. Low educational level was associated with frailty using the Fried’s phenotype model. More studies investigating the risk factors of frailty using different screening tools are needed, in order to further enhance our understanding of the risk of frailty among older adults and to design appropriate interventions.

## Figures and Tables

**Table 1:** Demographics of the participants according to their frail status (*n* = 205).

	Robust Health	Pre-Frail	Frail
	n (%)/Mean ± SD	n (%)/Mean ± SD	n (%)/Mean ± SD
**5-item FRAIL**			
*n* (%)	105 (51.22)	54 (26.34)	46 (22.43)
Age, mean (SD), years	70.8 ± 5.6	71.0 ± 5.1	71.9 ± 5.3
BMI, mean (SD), kg/m^2^	26.0 ± 4.1	25.7 ± 3.4	26.6 ± 4.4
Gender, Male, *n* (%)	56 (53.3)	21 (38.9)	18 (39.1)
Education (low), *n* (%)	33 (31.4)	32 (59.3)	23 (50)
Living alone, *n* (%)	19 (18.1)	9 (16.7)	9 (19.6)
Smoking, *n* (%)	6 (5.7)	2 (3.7)	1 (2.2)
Consuming alcohol, *n* (%)	13 (12.4)	3 (5.6)	2 (4.3)
Polypharmacy, *n* (%)	1 (1.0)	9 (16.7)	19 (41.3)
Comorbidity			
0	14 (13.3)	4 (7.4)	3 (6.5)
1	66 (62.9)	27 (50.0)	19 (41.3)
2	25 (23.8)	23 (42.6)	24 (52.2)
**CHS_PCF**			
*n* (%)	38 (18.53)	73 (35.61)	94 (45.85)
Age, mean (SD), years	69.7 ± 3.6	70.4 ± 5.3	72.1 ± 5.8
BMI, mean (SD), kg/m^2^	25.6 ± 3.9	25.9 ± 3.9	26.4 ± 4.1
Gender, Male, *n* (%)	19 (50.0)	30 (41.1)	46 (48.9)
Education (low), *n* (%)	8 (21.1)	30 (41.1)	50 (53.2)
Living alone, *n* (%)	7 (18.4)	17 (23.3)	13 (13.8)
Smoking, *n* (%)	1 (2.6)	4 (5.5)	4 (4.3)
Consuming alcohol, *n* (%)	4 (10.5)	7 (9.6)	7 (7.4)
Polypharmacy, *n* (%)	1 (2.6)	2 (2.7)	26 (27.7)
Comorbidity			
0	4 (10.5)	11 (15.1)	6 (6.4)
1	24 (63.2)	47 (64.4)	41 (43.6)
2	10 (26.3)	15 (20.5)	47 (50.0)
**SOF**			
*n* (%)	138 (67.31)	54 (26.34)	13 (6.341)
Age, mean (SD), years	70.8 ± 5.2	71.4 ± 5.9	72.3 ± 5.5
BMI, mean (SD), kg/m^2^	26.0 ± 4.2	26.1 ± 3.2	26.2 ± 4.1
Gender, Male, *n* (%)	67 (48.6)	23 (42.6)	5 (38.5)
Education (low), *n* (%)	52 (37.7)	29 (53.7)	7 (53.8)
Living alone, *n* (%)	25 (18.1)	10 (18.5)	2 (15.4)
Smoking, *n* (%)	6 (4.3)	3 (5.6)	0 (0.0)
Consuming alcohol, *n* (%)	15 (10.9)	3 (5.6)	0 (0.0)
Polypharmacy, *n* (%)	15 (10.9)	9 (16.7)	5 (38.5)
Comorbidity			
0	16 (11.6)	3 (5.6)	2 (15.4)
1	79 (57.2)	27 (50.0)	6 (46.2)
2	43 (31.2)	24 (44.4)	5 (38.5)

FRAIL, Fatigue, Resistance, Ambulation, Illness, and Loss of Weight. CHS_PCF, Cardiovascular Health Phenotypic Classification of Frailty. SOF, Study of Osteoporotic Fracture. SD, standard deviation. BMI, body mass index. Comorbidity: 0, no disease; 1, one to two diseases; 2, three or more diseases. Disease category: hypertension, diabetes mellitus, hyperlipidemia, cerebral vascular disease, cardiovascular disease, pulmonary disease, liver disease, urologic disease, neurology disease, and malignant cancer.

**Table 2:** Regression model of the 5-item Fatigue, Resistance, Ambulation, Illness, and Loss of Weight scale.

	Crude (95% CI)	*p*-Value	Adjusted (95% CI) *	*p*-Value	Adjusted (95% CI) †	*p*-Value
Age	1.04 (0.98–1.10)	0.220				
BMI	1.05 (0.96–1.13)	0.275				
Gender (male)	0.68 (0.35–1.34)	0.267				
Education (high)	0.69 (0.36–1.34)	0.272				
Live alone	1.14 (0.49–2.62)	0.762				
Smoke	0.42 (0.05–3.44)	0.419				
Alcohol	0.41 (0.09–1.84)	0.242				
Polypharmacy	10.49 (4.40–24.99)	<0.001	9.12 (3.6–23.16)	<0.001	9.02 (3.32–26.16)	<0.001
Comorbidity						
0	1.00		1.00			
1–2	1.23 (0.33–4.58)	0.762	0.98 (0.26–3.73)	0.9819		
>2	3.00 (0.80–11.19)	0.102	1.35 (0.34–5.44)	0.6708		
Per category	2.07 (1.18–3.64)	0.012			1.24 (0.63–2.46)	0.603
Goodness-of-fit statistic *			χ^2^ = 0.30, *p* = 0.859			

Crude odds ratio (OR) was calculated using the logistic regression model. * Adjusted polypharmacy and comorbidity; the Hosmer–Lemeshow goodness-of-fit test was used. † Adjusted OR was calculated using the exact logistic regression model. Comorbidity: 0, no disease; 1, one to two diseases; 2, three or more diseases.

**Table 3:** Regression model of Cardiovascular Health Phenotypic Classification of Frailty (CHS_PCF) index.

	Crude (95% CI)	*p*-Value	Adjusted (95% CI) *	*p*-Value	Adjusted (95% CI) †	*p*-Value
Age	1.07 (1.02–1.13)	0.010	1.06 (0.99–1.12)	0.058	1.06 (0.99–1.12)	0.054
BMI	1.04 (0.97–1.11)	0.330				
Gender (male)	1.21 (0.70–2.10)	0.493				
Education (high)	0.46 (0.26–0.80)	0.007	0.50 (0.26–0.96)	0.036	0.45 (0.23–0.87)	0.015
Live alone	0.58 (0.28–1.22)	0.151				
Smoke	0.94 (0.25–3.61)	0.931				
Alcohol	0.73 (0.27–1.97)	0.536				
Polypharmacy	13.76 (4.01–47.23)	<0.001	8.98 (2.51–32.11)	<0.001	8.81 (2.42–48.81)	<0.001
Comorbidity						
0	1.00		1.00			
1	1.44 (0.52–4.01)	0.481	1.32 (0.46–3.83)	0.605		
2	4.70 (1.62–13.62)	0.004	3.28 (1.06–10.15)	0.034		
Per category	2.57 (1.59–4.18)	<0.001			2.02 (1.17–3.56)	0.001
Goodness-of-fit statistic *			χ^2^ = 3.03, *p* = 0.882			

Crude odds ratio (OR) was calculated using the logistic regression model. * Adjusted age, education, polypharmacy, and comorbidity; the Hosmer–Lemeshow goodness-of-fit test was used. † Adjusted OR was calculated using the exact logistic regression model. Comorbidity: 0, no disease; 1, one to two diseases; 2, three or more diseases.

**Table 4:** Regression model of Study of Osteoporotic Fracture (SOF) scale.

	Crude (95% CI)	*p*-Value	Adjusted (95% CI) *	*p*-Value	Adjusted (95% CI) †	*p*-Value
Age	1.04 (0.95–1.15)	0.395				
BMI	1.01 (0.87–1.16)	0.929				
Gender (male)	0.71 (0.22–2.24)	0.558				
Education (high)	0.63 (0.2–1.93)	0.415				
Live alone	0.82 (0.17–3.84)	0.797				
Polypharmacy	4.38 (1.32–14.47)	0.016	6.10 (1.47–25.3)	0.013	6.31 (1.21–33.29)	0.027
Comorbidity						
0	1.00		1.00			
1	0.54 (0.1–2.87)	0.467	0.43 (0.08–2.35)	0.326		
2	0.71 (0.13–3.95)	0.695	0.29 (0.04–2.12)	0.225		
Per category	0.95 (0.39–2.33)	0.915			0.57 (0.18–1.78)	0.396
Goodness-of-fit statistic *			χ^2^ = 0.23, *p* = 0.892			

Crude odds ratio (OR) was calculated using the logistic regression model. * Adjusted polypharmacy and comorbidity; the Hosmer–Lemeshow goodness-of-fit test was used. † Adjusted OR was calculated using the exact logistic regression model. Comorbidity: 0, no disease; 1, one to two diseases; 2, three or more diseases.

## Data Availability

The data presented in this study are available on request from the corresponding author. The data are not publicly available due to privacy issues.
